# Transcriptome and metabolome analyses of Shatian pomelo (*Citrus grandis* var. Shatinyu Hort) leaves provide insights into the overexpression of the gibberellin-induced gene *CcGASA4*

**DOI:** 10.3389/fpls.2022.1022961

**Published:** 2022-11-03

**Authors:** Tianli Wu, Kaidong Liu, Min Chen, Bo Jiang, Qijing Gong, Yun Zhong

**Affiliations:** ^1^ Life Science and Technology School, Lingnan Normal University, Zhanjiang, China; ^2^ Institute of Fruit Tree Research, Guangdong Academy of Agricultural Sciences, Guangzhou, China; ^3^ Key Laboratory of South Subtropical Fruit Biology and Genetic Resource Utilization (MOA), Guangzhou, China; ^4^ Key Laboratory of Tropical and Subtropical of Fruit Tree Research, Science and Technology Department of Guangdong Province, Guangzhou, China

**Keywords:** *Citrus*, gibberellin, *CcGASA4*, transcriptome, metabolome, hormone, plant–pathogen interaction

## Abstract

The gibberellic acid (GA)-stimulated* Arabidopsis* (*GASA*) gene family is highly specific to plants and plays crucial roles in plant growth and development. *CcGASA4* is a member of the GASA gene family in citrus plants; however, the current understanding of its function in citrus is limited. We used CcGASA4-overexpression transgenic citrus (OEGA) and control (CON) plants to study the role of *CcGASA4* in Shatian pomelo. The RNA sequencing (RNA-seq) analysis showed that 3,522 genes, including 1,578 upregulated and 1,944 downregulated genes, were significantly differentially expressed in the CON versus OEGA groups. The Gene Ontology enrichment analysis showed that 178 of the differentially-expressed genes (DEGs) were associated with flowers. A Kyoto Encyclopedia of Genes and Genomes (KEGG) enrichment analysis showed that the DEGs were enriched in 134 pathways, including “plant–pathogen interaction”, “MAPK signaling pathway-plant”, “phenylpropane biosynthesis”, “plant hormone signal transduction”, “phenylalanine, tyrosine and tryptophan biosynthesis”, and “flavonoid and flavonol biosynthesis”. The most significantly-enriched pathway was “plant–pathogen interaction”, in which 203 DEGs were enriched (126 DEGs were upregulated and 78 were downregulated). The metabolome analysis showed that 644 metabolites were detected in the OEGA and CON samples, including 294 differentially-accumulated metabolites (DAMs; 83 upregulated versus 211 downregulated in OEGA compared to CON). The metabolic pathway analysis showed that these DAMs were mainly involved in the metabolic pathways of secondary metabolites, such as phenylpropanoids, phenylalanine, flavone, and flavonol biosynthesis. Thirteen flavonoids and isoflavones were identified as DAMs in OEGA and CON. We also discovered 25 OEGA-specific accumulated metabolites and found 10 that were associated with disease resistance. *CcGASA4* may therefore play a functional role in activating the expression of MAPK signaling transduction pathway and disease resistance genes, inhibiting the expression of auxin- and ethylene-related genes, and activating or inhibiting the expression of brassinosteroid biosynthesis- and abscisic acid-related genes. *CcGASA4* may also play a role in regulating the composition and abundance of flavonoids, isoflavones, amino acids, purines, and phenolic compounds. This study provides new insights into the molecular mechanisms of action of *CcGASA4* in citrus plants.

## Introduction

Gibberellic acid-stimulated *Arabidopsis* (GASA) proteins comprise a class of cysteine-rich small molecule polypeptides that are widely distributed and contain a large number of coding genes, most of which are regulated by gibberellins (Wigoda et al., 2006; Zhang and Wang, 2008). GASA family members were first identified in tomato (Shi et al., 1992), and numerous GASA homologs have been identified in various plant species, such as *Arabidopsis* (Chen et al., 2007), rice (Muhammad et al., 2019), and *Fragaria ananassa* (de la Fuente et al., 2006). Typical GASA proteins have low molecular weights and are composed of 80–270 amino acids. GASA usually has three different domains: a putative *N*-terminal signaling peptide of 18–29 amino acids, highly-variable region (7–31 amino acids) with a large difference in both amino acid composition and sequence length, and conserved *C*-terminal GASA domain of approximately 60 amino acids (including 12 cysteine residues) (Aubert et al., 1998). A few GASA proteins contain a proline-rich protein (PRP) domain in addition to a GASA domain, such as PRGL of *Gerbera hybrida* (Peng et al., 2010) and AtGASA14 of *Arabidopsis* (Sun et al., 2013). The GASA domain is the core domain of GASA proteins and is present in all members of the protein family. Deletions of the GASA domain or mutations in the conserved cysteine residues in the domain can cause the loss of protein function. These conserved cysteine residues are therefore necessary for maintaining the structure and function of GASA proteins (Rubinovich and Weiss, 2010; Sun et al., 2013). Cysteine pairs are separated by several amino acids to form catalytic disulfide bonds (redox-active cysteines) (Buchanan and Balmer, 2005). Therefore, the 12 cysteine residues in the GASA domain may contribute to the biochemical stability of the protein structure. Studies have shown that disulfide bonds are also key to the interactions between GASA proteins and other proteins. For example, the Snakin-2 protein of French bean and PRPs form a protein complex (approximately 42 kDa; FBCBP) *via* disulfide bond oxidation, which is further involved in plant defense processes (Bindschedler et al., 2006).

The GASA family of proteins is involved in a diverse range of functions, including plant development, hormonal crosstalk, and defense. *GASA* genes are thought to be involved in various developmental processes in plants, such as lateral root initiation and development, leaf expansion, flower induction, corolla and carpel cell expansion, the cessation of petal cell elongation, the reduction of silique elongation, fruit cell size regulation, cell division, seed development, and germination (Aubert et al., 1998; Kotilainen et al., 1999; Rubinovich and Weiss, 2010; Trapalis et al., 2017). Most *GASA* genes are also involved in hormone signaling pathways, such as the gibberellin, abscisic acid (ABA), and auxin pathways (Furukawa et al., 2006; Li et al., 2011). For example, rice *GSR1*, a GA-induced and brassinosteroid (BR)-repressed gene, mediates the interaction between the BR and GA pathways by directly interacting with the BR biosynthetic enzyme DIM/DWF1 (Wang et al., 2009). In *Arabidopsis*, *GASA6* is upregulated by growth hormones (auxin, BR, cytokinin, and GA) and downregulated by stress hormones (ABA, JA, and salicylic acid [SA]), thus playing a role in hormonal crosstalk (Qu et al., 2016). The Beechnut gene *FsGASA4* modulates SA responses by activating the genes involved in SA biosynthesis and action (Alonso-Ramírez et al., 2009). *AtGASA5* was induced by ABA and repressed by GA (Zhang and Wang, 2008). The SA signaling pathway appeared to be blocked in *AtGASA5* gain-of-function plants, the phenotype of which can be partially rescued by applying SA externally (Zhang and Wang, 2011).

The biochemical activity and defense functions of GASA proteins have also been partially studied. For example, an expression analysis of *GIP2*, *GIP4*, and *GIP5* indicated that these genes are induced by H_2_O_2_ and may be involved in redox regulation (Wigoda et al., 2006). The expression of the potato homologs *Snakin‐1* and *Snakin‐2* responded to wounding and pathogenic infections (Segura et al., 1999; Berrocal-Lobo et al., 2002). The *Arabidopsis* gene *GASA14* is involved in abiotic stress (ABA and salt) tolerance by regulating the accumulation of reactive oxygen species (ROS) (Sun et al., 2013). The overexpression of *AtGASA4* in *Arabidopsis* has been found to inhibit the accumulation of ROS, and transgenic seeds are partially resistant to treatment with the NO donor sodium nitroprusside (Rubinovich and Weiss, 2010). The overexpression of *AtGASA4*, which is also involved in light signaling, improves the heat tolerance of transgenic *Arabidopsis* (Chen et al., 2007; Ko et al., 2007). In contrast, *AtGASA5* negatively regulates heat tolerance and the GA response (Zhang et al., 2009; Rubinovich et al., 2014).

Our previous studies found that the citrus gene *CcGASA4* is induced by the *Citrus tristeza* virus (CTV) (Wu et al., 2020). Similar to all of the other GASA proteins identified thus far, the CcGASA4 protein contains a well-conserved domain carrying putative redox-active cysteine residues. Therefore, we speculate that the CcGASA4 protein may play an essential role in the regulation of growth, development, and disease resistance in citrus plants. In this study, we obtained CcGASA4-overexpressing transgenic plants through genetic transformation and investigated the function of *CcGASA4* in citrus plants. To the best of our knowledge, this is the first study to analyze the function of *CcGASA4* in Shatian pomelo through metabolomic and transcriptomic analyses. Our findings provide insights into CcGASA4-mediated metabolic regulation and may contribute to accelerating the breeding of *Citrus* species.

## Materials and methods

### Creation of the CcGASA4-overexpression vector

The *Citrus clementina* cultivar was provided by the Institute of Fruit Tree Research from the Guangdong Academy of Agricultural Sciences and was used for RNA isolation and *CcGASA4* gene amplification. The *CcGASA4* coding sequence was cloned as described in a previous study (Wu et al., 2020). To construct the overexpression vector, entry clone fragments were inserted into the destination pFGC5941 vector using the *Asc*I and *Sma*I restriction sites (**Figure S1**) under the control of a 35S promoter through a homologous recombination reaction using the Trelief™ SoSoo cloning kit (Tsingken, China). The recombination reaction solution was transformed into DH5α cells, and resistant monoclonal colonies were detected by PCR using the primers 5941F2624 and 5941R4286. The *CcGASA4-pFGC5941* plasmid was extracted from the selected positive incubation solution using the HiPure Plasmid EF Mini Kit (Magen, China) and confirmed by sequencing. The constructs were transformed into *Agrobacterium* EHA105 cells using heat shock. Transformation was confirmed by PCR with primers 35SF/GASA4R, and the successful transformant was named CcGASA4-pFGC5941-105. All the primers used in this study are listed in **Supplementary Table S1**.

### Generation of transgenic citrus lines

The CcGASA4-overexpression vector was transformed into Shatian pomelo using the *Agrobacterium*-mediated hypocotyl genetic transformation method (Wu et al., 2015). The expression level of the *CcGASA4* gene in CcGASA4-overexpression transgenic citrus (OEGA) was detected using qRT-PCR. Total RNA was extracted from mature 3-year-old leaves of healthy OEGA plants using a polysaccharide and polyphenol plant total RNA rapid extraction kit (Bioteke, China), following the manufacturer’s instructions. The quality and quantity of the total RNA were analyzed using a NanoDrop 2000C (Thermo Fisher Scientific, MA, USA). qRT-PCR experiments were performed according to the method described by Wu et al. (2021). The data were statistically analyzed using an analysis of variance(ANOVA). Duncan’s LSD multiple range test (*P* ≤ 0.05) was used to determine significant changes. The relative expression levels of the genes were determined using Origin 2019b.

### Transcriptome analysis and qRT-PCR validation of differentially-expressed genes in RNA-seq

For the transcriptome profiling and analysis, RNA isolation and purification, cDNA library construction, and sequencing were performed by the commercial biotech company Metware Biotechnology Co., Ltd. (https://www.metware.cn/; Wuhan, China). In brief, total RNA was isolated from the OEGA leaves and used to prepare RNA-seq libraries. The libraries were sequenced using the Illumina HiSeq platform. Clean reads were obtained by passing the raw sequencing data through quality control (QC) procedures. The reads were then mapped to the pomelo (*Citrus grandis*) genome sequence (http://citrus.hzau.edu.cn/orange/) (Xu et al., 2013) using the HISAT2 software (Kim et al., 2015). To obtain a comprehensive view of the gene expression profile associated with CcGASA4, we used DESeq2 to identify the DEGs). Reads per kilobase per million mapped reads (FPKM) values were used as indicators for gene and transcript level quantification (Wang et al., 2015). The DEGs were determined using the following filtering parameters: false discovery rate (FDR) < 0.05 and |log_2_foldchange| ≥ 1. All of the DEGs were analyzed using Gene Ontology (GO) (Ashburner et al., 2000) and Kyoto Encyclopedia of Genes and Genomes (KEGG) enrichment analyses with the KOBAS software (Kanehisa et al., 2007). To confirm the reliability of the RNA-seq data, the important genes of the “phenylalanine, tyrosine and tryptophan biosynthesis”, “plant–pathogen interaction”, and “flavone and flavonol biosynthesis” pathways were screened from the RNA-seq annotation library. Seventeen genes were selected for a qRT-PCR analysis. qRT-PCR was performed as previously described by Wu et al. (2021).

### Widely-targeted metabolic analysis of the metabolites in the citrus samples

Metabolome profiling was performed by Metware Biotechnology Co., Ltd. The extraction and separation of the metabolites from the OEGA and control (CON) leaves were performed as previously reported (Wang et al., 2020). In brief, 100 mg of powder per sample were extracted overnight at 4°C with 1.2 mL of 70% aqueous methanol. The samples were then centrifuged at 12,000 g for 10 min and filtered through a 0.22-μm micropore filter membrane to obtain leaf extracts. The extracts were analyzed using a UPLC-ESI-MS/MS system (UPLC, SHIMADZU Nexera X2, www.shimadzu.com.cn/; MS, Applied Biosystems 4500 QTRAP, www.appliedbiosystems.com.cn/). Qualitative and quantitative analyses of the metabolites were performed using electrospray ionization (ESI)-quadrupole ion trap (QTRAP)-tandem mass spectrometry (MS/MS) based on the self-built database Metware in-house MS2 spectral tag library (Metware database, MWDB).

The analytical conditions were as follows. A UPLC column (Agilent SB-C18 [1.8 µm, 2.1 mm*100 mm]) was used. The mobile phase consisted of solvent A, which comprised pure water with 0.1% formic acid, and solvent B, which comprised acetonitrile with 0.1% formic acid. Sample measurements were performed using a gradient program that employed the starting conditions of 95% A and 5% B. Within 9 min, a linear gradient of 5% A and 95% B was programmed, and compositions of 5% A and 95% B were maintained for 1 min. Compositions of 95% A and 5% B were then adjusted within 1.1 min and maintained for 2.9 min. The column oven temperature was set to 40°C. The injection volume was 4 μL. The effluent was alternatively connected to an ESI-QTRAP-MS system for scanning detection.

LIT and triple quadrupole (QQQ) scans were acquired on a triple QTRAP mass spectrometer (AB4500 QTRAP UPLC/MS/MS System) equipped with an ESI Turbo Ion-Spray interface, which operated in both positive and negative ion modes and was controlled by Analyst 1.6.3 software (AB Sciex). The ESI source operation parameters were as follows: ion source, turbo spray; source temperature, 550°C; ion spray voltage (IS), 5500 V (positive ion mode)/–4500 V (negative ion mode); ion source gas I, gas II, and curtain gas were set to 50, 60, and 25.0 psi, respectively; and collision gas, high. Instrument tuning and mass calibration were performed using 10 and 100 μmol/L polypropylene glycol solutions in the QQQ and LIT modes, respectively. QQQ scans were acquired as multiple reaction monitoring (MRM) experiments with a collision gas (nitrogen) set to the medium. Declustering potential (DP) and collision energy (CE) for the individual MRM transitions were performed with further DP and CE optimizations. A specific set of MRM transitions was monitored for each period according to the metabolites eluted within the period.

### Statistical analysis

A principal component analysis (PCA) of the metabolites was performed using the statistical function prcomp in the software R (www.r-project.org). The metabolite content data were normalized using unit variance scaling. The accumulation mode of the metabolites among the different samples was subjected to a hierarchical cluster analysis (HCA), and heatmaps were drawn using the pheatmap package in R. The HCA results of the samples and metabolites were presented as heatmaps with dendrograms, whereas Pearson’s correlation coefficients (PCC) between the samples were calculated by the cor function in R and presented as heatmaps. An orthogonal partial least squares discriminant analysis (OPLS-DA), which combines orthogonal signal correction and a partial least squares discriminant analysis (PLS-DA), was used to screen the different variables. To identify the differentially-accumulating metabolites (DAMs), we first selected the metabolites with a foldchange (FC) ≥ 2 (upregulated) or FC ≤ 0.5 (downregulated) in the OEGA sample compared to the CON. We then screened the DAMs using a threshold variable importance in projection (VIP) value of ≥ 1 from the OPLS-DA model. The VIP values were extracted from the OPLS-DA results, which also contained score and permutation plots, and were generated using the R package MetaboAnalyst R. The data were log-transformed (log_2_) and mean-centered before the OPLS-DA. To assess the significance of the differences in the abundance of metabolites, a two-tailed Student’s *t*-test was used to compare individual lines with their relevant controls. The identified metabolites were annotated and mapped to metabolic pathways using the KEGG database (http://www.kegg.jp) (Kanehisa and Goto, 2000) to determine their associated pathways. A pathway enrichment analysis was performed on the web-based server Metabolite Sets Enrichment Analysis (MSEA; http://www.msea.ca), and their significance was determined using hypergeometric test *P* values. Pathways with Bonferroni-corrected *P* values < 0.05 were considered significantly enriched.

## Results

### *CcGASA4* genetic transformation and generation of the *CcGASA4*-overexpression transgenic citrus lines

**Figure 1:**
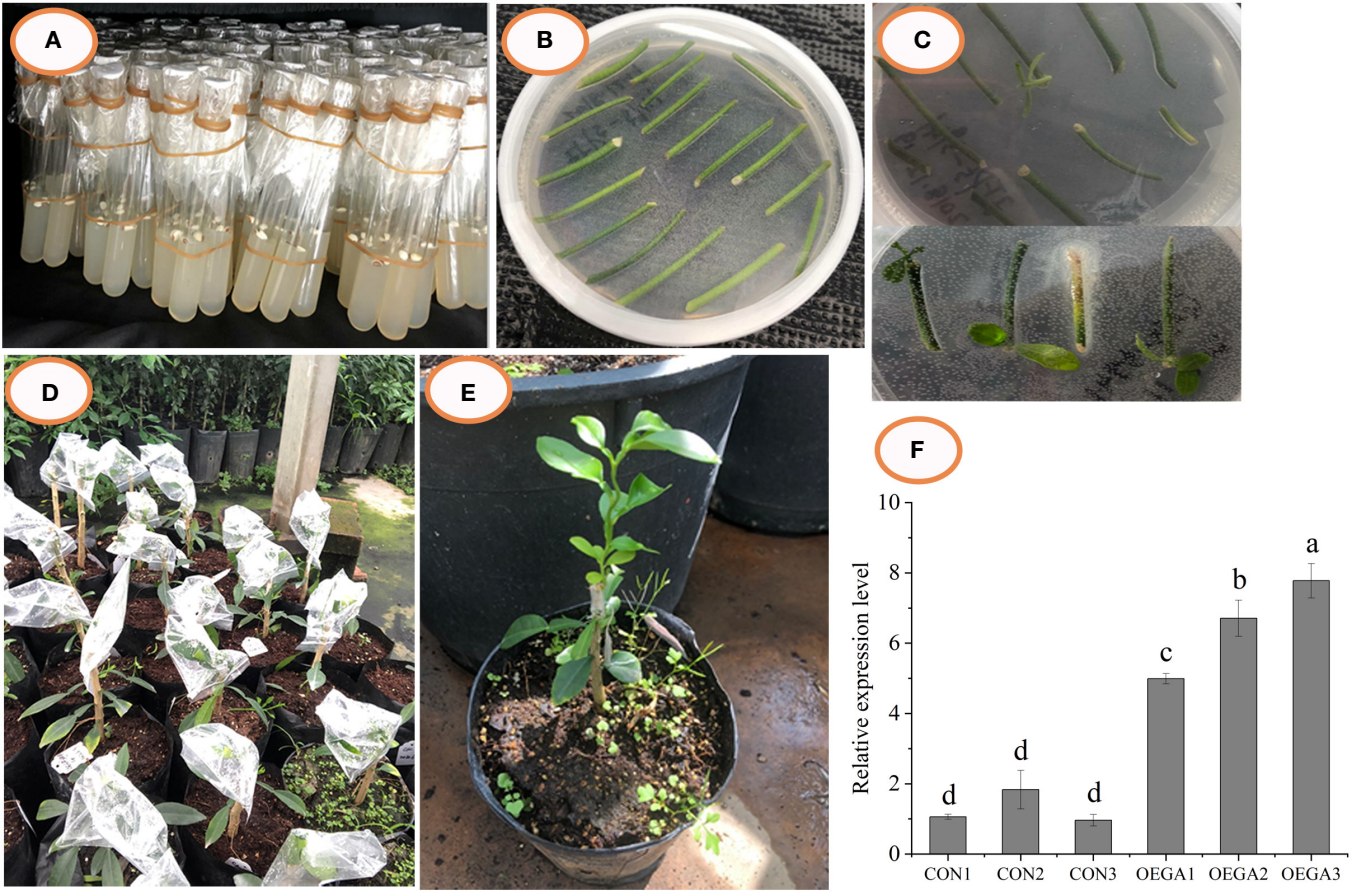
Transgenic citrus plants overexpressing CcGASA4 were obtained through genetic transformation. **(A–E)** Process of *Agrobacterium*-mediated *CcGASA4* genetic transformation. **(A)** citrus seeds were sown on MS medium; **(B)** dark culture; **(C)** light culture; **(D)** grafted resistant buds on rootstocks; **(E)** surviving resistant buds. **(F)** qRT-PCR analysis of *CcGASA4* expression in the OEGA lines. Data are presented as the mean ± standard error (SE) of three qRT-PCR experiments. Different lowercase letters (a–d) on the bars indicate statistically-significant differences (*P* < 0.05) based on Duncan’s LSD multiple range test.

The *CcGASA4* sequence was successfully cloned from *Citrus clementina* and placed under the control of a 35S promoter to obtain a *CcGASA4-pFGC5941* overexpression vector (**Figure S1B, C**). Transformants were produced through the *Agrobacterium*-mediated genetic transformation of citrus (**Figures 1A–E**). The detection of the genetically-transformed plants by PCR showed that there were three transgenic-positive plants (**Figure S1D**). The expression analysis showed that the expression level of *CcGASA4* in the OEGAs was higher than that in the CONs. Its relative expression level in OEGA3 was the highest, being approximately eight times higher than that in the CON plants (**Figure 1F**). Therefore, we selected three grafted OEGA3 lines as experimental materials for the transcriptome and metabolome analyses.

### Overview of the transcriptome analysis

**Figure 2:**
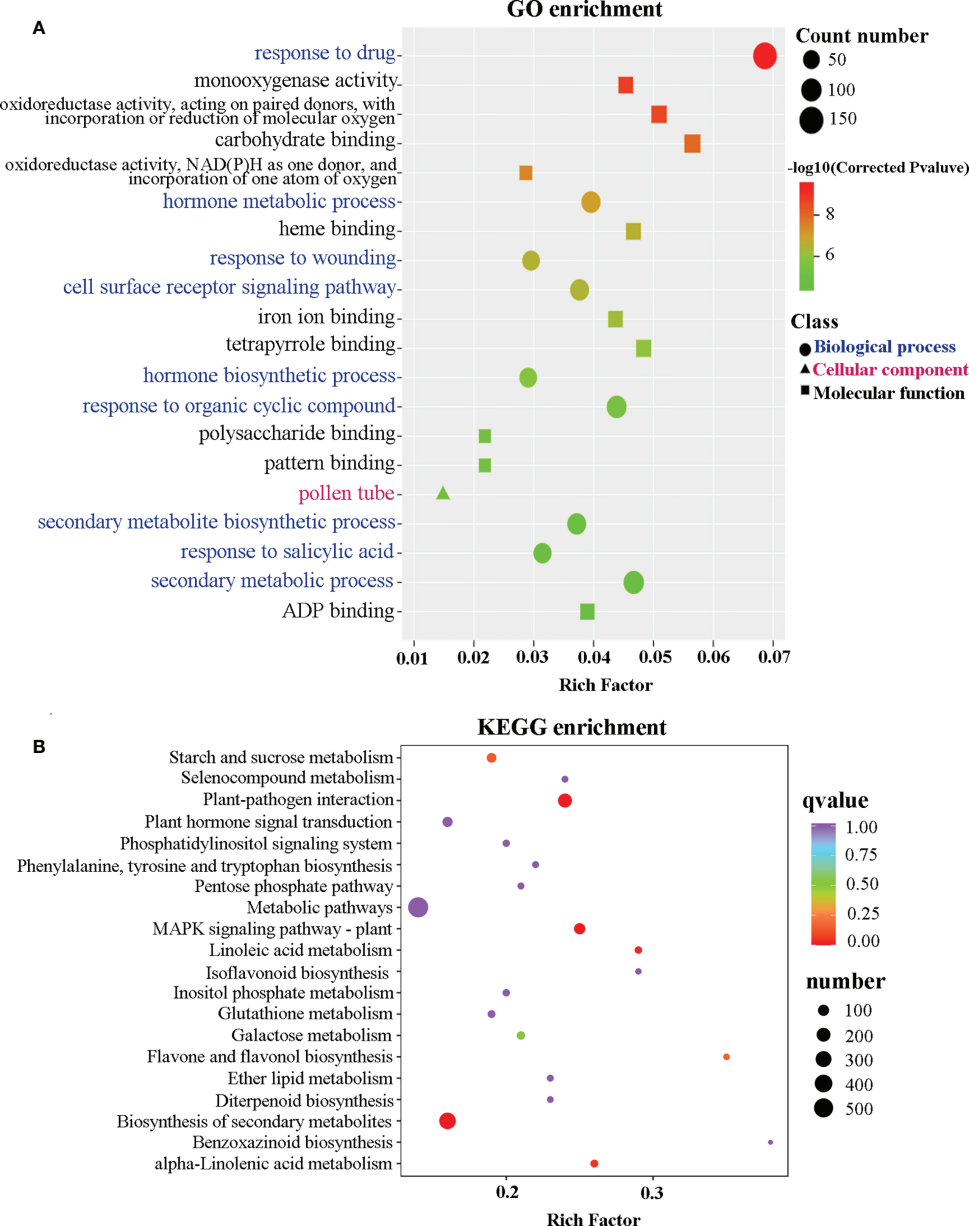
Scatter plot of the GO and KEGG enrichment analyses based on the DEGs. **(A)** Scatter plot of the GO enrichment analysis based on the DEGs. The ordinate represents a GO term. The abscissa indicates a rich factor. The greater the rich factor, the greater the degree of enrichment. The larger the point, the greater the number of DEGs enriched in the GO pathway. The redder the color of the dot, the more significant the enrichment. **(B)** Scatter plot of the DEGs enriched in the KEGG pathways. The ordinate indicates the KEGG pathway. The abscissa indicates a rich factor. The greater the rich factor, the greater the degree of enrichment. The larger the point, the greater the number of DEGs enriched in the KEGG pathway. The redder the color of the dot, the more significant the enrichment.

To further understand the role of *CcGASA4* in citrus Shatian pomelo, an RNA-seq analysis was performed. A total of 38.74 Gb of clean reads and approximately 6 Gb of clean data per sample were generated from six biological samples, including three OEGA and three CON samples. The average Q30 value of the raw reads was 94.52%, and 94.44% of the reads were mapped to the *Citrus grandis* genome sequences (**Table S2**). The RNA-seq results showed that 3,522 genes, including 1,578 upregulated and 1,944 downregulated genes, were significantly differentially expressed in the CON versus OEGA groups (**Table S3**). Based on the GO annotations, all of the DEGs were grouped into three major categories: molecular functions, cellular components, and biological processes. The enrichment analysis indicated that the DEGs were mainly enriched in the “response to drug”, “carbohydrate binding”, “oxidoreductase activity, acting on paired donors, with incorporation or reduction of molecular oxygen”, “defense response to bacterium”, “immune system process”, “response to abscisic acid”, “cell wall organization”, “regulation of response to stress”, “hormone metabolic process”, “response to wounding”, and “pollen tube” GO terms (**Figure 2A**, **Table S4**). The GO enrichment analysis further showed 178 DEGs that were related to flower development, and there were 104 genes related to pollen development, of which 61 genes were upregulated and 43 genes were downregulated (**Table S5**).

To better understand the pathways activated by the overexpression of *CcGASA4*, a KEGG enrichment analysis was performed, which assigned 1,241 DEGs to 134 KEGG pathways (**Table S6**, **Figure S2**). Among these pathways, “metabolic pathways”, “biosynthesis of secondary metabolites”, “plant–pathogen interaction”, “MAPK signaling pathway-plant”, “phenylpropanoid biosynthesis”, “protein processing in endoplasmic reticulum”, “plant hormone signal transduction”, “phenylalanine, tyrosine and tryptophan biosynthesis”, “flavone and flavonol biosynthesis”, and “starch and sucrose metabolism” were significantly enriched (**Figure 2B**). In addition to the “metabolic pathways” and “biosynthesis of secondary metabolites” pathways, the DEGs enriched in the “plant–pathogen interaction” pathway was the most significant. They comprised 203 DEGs, of which 126 were upregulated and 78 were downregulated.

**Figure 3:**
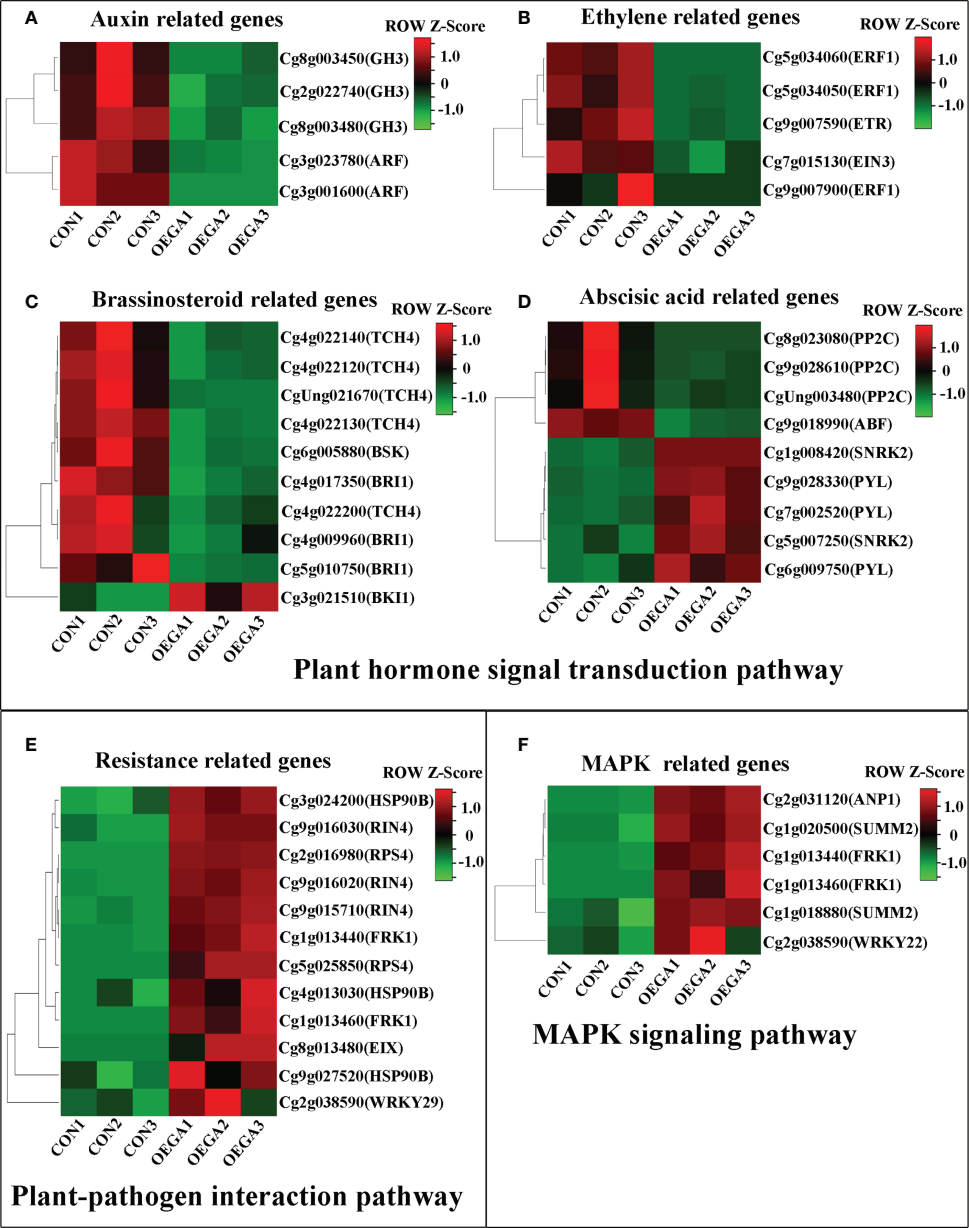
Heatmap of the DEGs in the OEGA and CON plants. **(A–D)** Heatmap of the plant hormone signal transduction pathway genes. **(E)** Heatmap of the plant–pathogen interaction pathway genes. **(F)** Heatmap of the MAPK pathway genes. The bar represents the scale of the expression levels of each gene (reads per kilobase per million mapped reads; FPKM) in the different samples, as indicated by the red and green rectangles. The metabolite production levels in the heatmaps were normalized by Z-scores. Genes in red indicate upregulation, whereas those in green indicate downregulation.

Heatmaps of the DEG subclusters were developed to better understand the key DEGs associated with the role of *CcGASA4.* Based on their functional annotations, the DEGs, including 29 plant hormone signal transduction pathway genes, 12 plant–pathogen interaction pathway genes, and six MAPK pathway genes, were shown in the heatmap (**Figure 3**; **Table S7**). These findings indicate that *CcGASA4* may play a functional role in activating the expression of MAPK signaling transduction pathway and disease resistance genes, inhibiting the expression of auxin- and ethylene-related genes, and activating or inhibiting the expression of BR biosynthesis- and ABA-related genes.

### Validation of candidate DEGs based on the qRT-PCR analysis

To confirm the DEGs obtained from the RNA-seq analysis, the expression levels of 17 candidate genes were randomly analyzed using qRT-PCR. These genes included six plant–pathogen interaction genes (*RPS4*, *RIN4*, *PTI6*, *RBOH*, *FRK1*, and *WRKY1*), six flavone and flavonol biosynthesis genes (two *IF7MaT* genes, two *C12RT1* genes, *CYP75B1*, and *FG2*), and five phenylalanine, tyrosine, and tryptophan biosynthesis genes (two *tyrAa* genes, two *pheA2* genes, and *trpB*) (**Figure S3**). The qRT-PCR results confirmed that the changes in the expression levels of all 17 genes were basically consistent with the RNA-seq data.

### Widely-targeted metabolome analysis of the OEGA transgenic citrus plants

**Figure 4:**
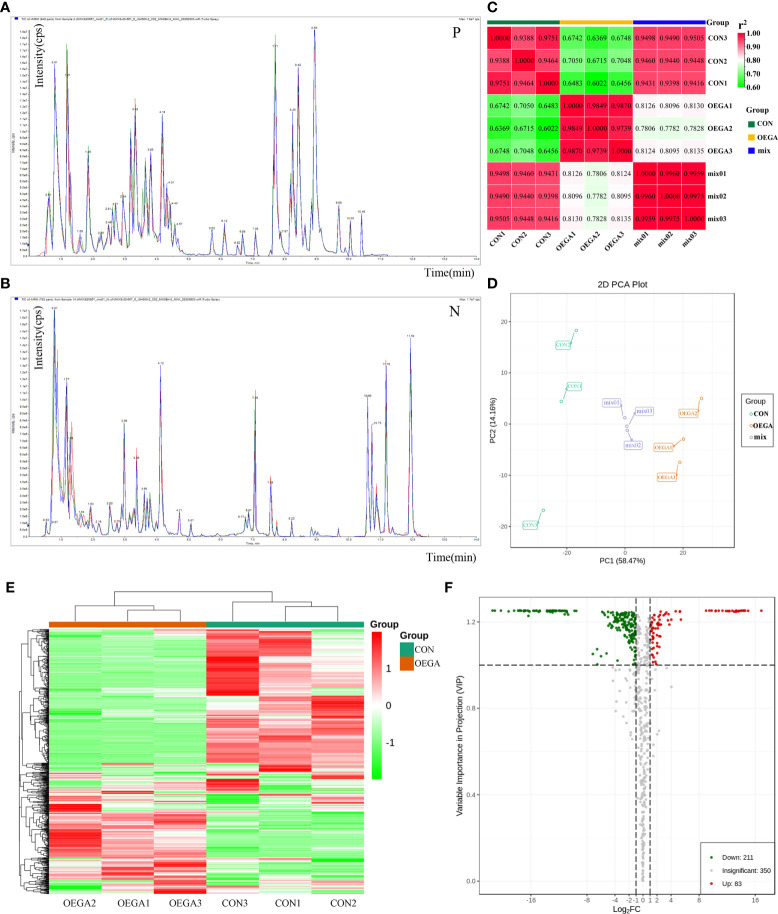
Metabolomics profiles of the citrus leaves from the OEGA and CON samples. **(A)** and **(B)** Overlapping analysis of the total ion current in the different quality control (QC) samples. The abscissa indicates the retention time (RT) of the metabolites, and the ordinate indicates the ion current intensity in counts per second (cps). The three different colors represent the three QC samples. N represents the negative ion mode, and P represents the positive ion mode. **(C)** Pearson’s correlation coefficients among the citrus samples CON, OEGA, and mix (QC). The abscissa and ordinate indicate corresponding sample names. The color represents the correlation coefficient (r^2^). **(D)** Principal component analysis of all the metabolites. OEGA: yellow; CON: green; mixed: purple. The abscissa represents the first principal component (PC1), and the ordinate represents the second principal component (PC2). The percentage indicates the interpretation rate of the principal components in the dataset. **(E)** Cluster heatmap of the differences in the metabolite content between the OEGA and CON groups. The sample name is indicated on the abscissa, and the metabolite information is shown on the ordinate. The cluster line on the left of the Figure is the metabolite cluster line, and that on the top of the Figure represents the sample cluster line. The different colors represent the values obtained after the relative content standardization process (red represents high content, and green represents low content). **(F)** Volcano plot of the differential metabolites. Green dots represent downregulated metabolites, red spots represent upregulated metabolites, and gray dots represent metabolites with insignificant differences.

To better understand the regulatory function of *CcGASA4* in the metabolite levels in citrus plants, we performed a widely-targeted metabolome analysis to reveal the metabolic changes between the OEGA and CON groups. The repeatability of metabolite extraction and detection was assessed using an overlapping analysis of the total ion current (TIC) in the different QC samples (**Figures 4A, B**). Three key parameters, including retention time, peak intensity, and the TIC curve, were evaluated and indicated that the detection signals of the samples were stable at different times. We further conducted a correlation analysis of the expression levels of metabolites based on PCC to determine the relationships between the biological replicates within the groups. The results indicated that all of the biological replicates exhibited similar expression patterns, thereby illustrating the high reliability of the sequencing data (**Figure 4C**). Thus, the sequencing quality was sufficient for further analysis.

In order to attain a preliminary understanding of the overall differences in the metabolites among the different groups, as well as the intra-group variations in the metabolites, we conducted a PCA on the samples, including the QC samples. The PCA was used to determine the contribution rates of the first two main components (PC1 and PC2), which scored 58.47% and 14.16%, respectively (**Figure 4D**). The PCA plot demonstrated that the three groups (OEGA, CON, and mixed) were clearly separated, but the three mixed samples were grouped together, indicating that they had similar metabolic profiles (**Figure 4D**). These data suggested that the samples were sufficiently reproducible to be suitable for a qualitative analysis.

To identify the DAMs and their various trends, we performed an HCA of all the detected metabolites and created cluster heatmaps. The metabolite profiles of the samples were divided into two main clusters, suggesting a clear variation in the abundance of the metabolites in the OEGA and CON samples (**Figure 4E**). The OPLS-DA model (foldchange score ≥ 2 or ≤ 0.5, VIP value > 1) further revealed 644 metabolites in all the samples, of which 294 metabolites displayed significantly-different levels between the two sample groups (**Table S8**). Volcano plots of the 294 DAMs showed that 83 and 211 metabolites were significantly accumulated in the OEGA and CON groups, respectively (**Figure 4F**). To visualize the class-specific patterns of the candidate metabolites, Z-score plots of the metabolites in the OEGA relative to the CON samples were generated (**Figure S4**).

**Figure 5:**
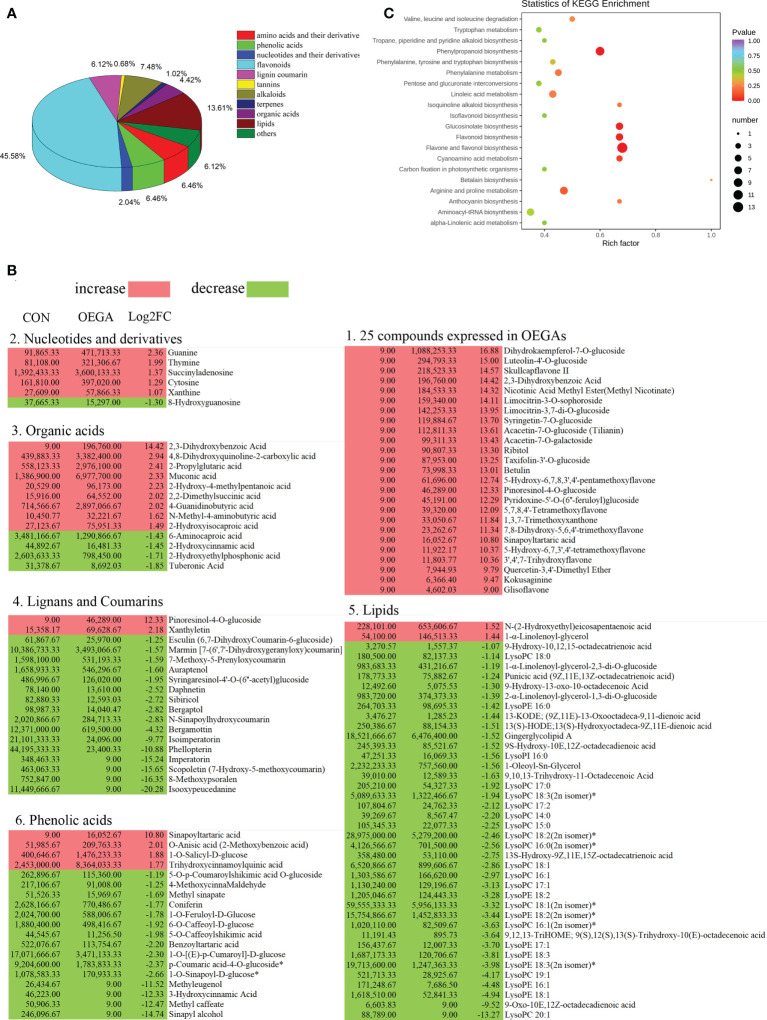
Pathway enrichment analysis of the differential metabolites in the OEGA transgenic citrus. **(A)** Classification and proportion of the differentially-accumulated metabolites. The numbers next to the pie chart represent the percentage of different types of DAMs among the total differential metabolites. **(B)** Abundance of the significant DAMs among the different metabolite classes. Red and green indicate increased and decreased levels of metabolites, respectively, as indicated by the colored squares. Lysophosphatidylcholine, LysoPC; lysophosphatidylethanolamine, LysoPE. **(C)** Significantly-enriched KEGG metabolic pathways. The abscissa indicates the enrichment factor, and the ordinate indicates the enrichment pathway. The dot sizes represent the number of differentially-enriched metabolites. The greater the rich factor, the greater the degree of enrichment. The larger the point, the greater the number of differentially-enriched metabolites in the pathway. The significance of the enrichment level is indicated by the color of the dot according to the color scheme shown on the right.

To further analyze the functional annotations of the DAMs and the enriched pathways that they are involved in, we mapped them to the KEGG database. A wide variety of metabolites, including 19 amino acids and their derivatives, 19 phenolic acids, six nucleotides and their derivatives, 134 flavonoids, 18 lignin coumarins, two tannins, 22 alkaloids, three terpenes, 13 organic acids, and 40 lipids, displayed considerable differences between the CON and OEGA groups (**Figure 5A**; **Table S9**). In the OEGA, 25 metabolites, including 16 flavonoids, two alkaloids, one organic acid, one terpenoid, one lignan and coumarin, one phenolic acid, and three other compounds, were at relatively-higher levels and did not accumulate considerably in the CON group (**Figure 5B**; **Table 1**). The significant OEGA-specific accumulated metabolites were the flavonoids dihydrokaempferol-7-O-glucoside (log_2_FC = 16.88) and luteolin-4’-O-glucoside (log_2_FC = 15) (**Figure 5B**). In addition, we detected six nucleic acid-related DAMs, five of which had accumulated with increased abundance. These included cytosine, thymine, guanine, xanthine, and succinyladenosine (**Figure 5B**). However, most of the differentially-accumulated lignans, coumarins, and lipids, such as isooxypeucedanine and lysoPC (20:1), were less abundant in the OEGA group (**Figure 5B**). The KEGG enrichment analysis showed that the DAMs were involved in 54 pathways (**Table S10**), including “flavonoids and flavonol biosynthesis” (16.666%), “phenylpropanoid biosynthesis” (11.538%), “flavonoid biosynthesis” (7.692%), “arginine and proline metabolism” (8.97%), “glucosinolate biosynthesis” (7.69%), and “biosynthesis of amino acids” (8.97%) (**Figure 5C**).

**Table 1:** 25 DAMs that were expressed in OEGA plants.

Index	Compounds	Class I	Description	Log2FC
Lmmn004625	Dihydrokaempferol-7-O-glucoside	Flavonoids	Flower-promoting activity at the extremely low concentration of 4.4×10^-9^ (Nakanishi et al., 1995); fruit development (Tian et al., 2006).	16.88
Hmpp003270	Luteolin-4'-O-glucoside	Flavonoids	Improved the symptoms of inflammation by decreasing the levels of interleukin-1β (IL-1β) and tumor necrosis factor-α (TNF-α) (Lin et al., 2018).	15.00
Zmhp004269	Skullcapflavone II	Flavonoids	A potential bradykinin antagonist, reduced the major pathophysiological features of allergic asthma, in part by acting on TGF-β1/Smad signaling pathways (Jang et al., 2012); attenuates ovalbumin-induced allergic rhinitis through the blocking of Th2 cytokine production and mast cell histamine release (Bui et al., 2017).	14.57
Hmcp001579	Limocitrin-3-O-sophoroside	Flavonoids	–	14.11
Hmcp001628	Limocitrin-3,7-di-O-glucoside	Flavonoids	–	13.95
HJAP006	Syringetin-7-O-glucoside	Flavonoids	It was first identified (Slimestad et al., 1999).	13.70
pmp000575	Acacetin-7-O-glucoside (Tilianin)	Flavonoids	Antimicrobial and anti-oxidant activities were carried out and significant results were obtained (Shen et al., 2004).	13.61
pmp000573	Acacetin-7-O-galactoside	Flavonoids	It was isolated as the anti-HIV compounds (Hu et al., 1994).	13.43
Xmsn002700	Taxifolin-3'-O-glucoside	Flavonoids	The compounds isolated from the genus Elephantorrhiza can be used in the treatment of benign prostatic hyperplasia (BPH). The extracts have been found to contain taxifolin-3′-O-glucoside (Fouche et al., 2015).	13.25
pmp000113	5-Hydroxy-6,7,8,3',4'-pentamethoxyflavone	Flavonoids	Estragon and thyme extracts showed potent inhibitory activities against chemical mediator release from rat basophilic leukemia RBL-2H3 cells. 5-hydroxy-6,7,8,3′,4′-pentamethoxyflavone was isolated from thyme as active components (Watanabe et al., 2005).	12.74
pmp000109	5,7,8,4'-Tetramethoxyflavone	Flavonoids	–	12.09
Zmhp004065	7,8-Dihydroxy-5,6,4'-trimethoxyflavone	Flavonoids	–	11.34
pmp000008	5-Hydroxy-6,7,3',4'-tetramethoxyflavone	Flavonoids	It could effectively protect hepatocyte against carbon tetrachloride (CCl4)-induced injury (Nam et al., 2005).	10.37
pmp000344	3',4',7-Trihydroxyflavone	Flavonoids	It has activity against Penicillium oxalicum and Trichoderma virens (Yang et al., 2018). It showed antibacterial activity (Dzotam et al., 2018). It can prevent apoptotic cell death in neuronal cells from hydrogen peroxide-induced oxidative stress (Kwon et al., 2015).	10.36
Lmcp005645	Quercetin-3,4'-Dimethyl Ether	Flavonoids	It enhances the expression of IL-1β gene through transcriptional regulation, which involves mechanisms that act downstream of the initial NF-κB and STAT (Lim et al., 2013).	9.79
pmp000362	Glisoflavone	Flavonoids	inhibitory effects of licorice phenolics on xanthine oxidase (Hatano et al., 1989).	9.00
pmn001378	Pinoresinol-4-O-glucoside	Lignans and Coumarins	The structure of this compound has been established (Casabuono and Pomillo, 1994).	12.33
mws0213	Ribitol	Others	Plays a role in abiotic responses in tomato (Almaghamsi et al., 2020).	13.30
pmb0803	Pyridoxine-5'-O-(6''-feruloyl) glucoside	Others	–	12.29
pmp001006	1,3,7-Trimethoxyxanthone	Others	1,3,7-trimethoxyxanthone is mutagenic to Salmonella typhimurium TA100 (Matsushima et al., 1985).	11.84
mws0146	Nicotinic Acid Methyl Ester (Methyl Nicotinate)	Alkaloids	–	14.32
HJAP159	Kokusaginine	Alkaloids	Mediates the inhibitory effect on the growth of human breast cancer cells and MDR resistant cells by inhibiting tubulin assembly (Chen et al., 2018); inhibit the proliferation of cancer cells and to induce a cell cycle arrest in a concentration-dependent manner in HeLa cells (Molnár et al., 2013).	9.47
pmp000438	Betulin	Terpenoids	Reduced cancer (NSCLC) cell colony-forming ability under normoxia (Kutkowska et al., 2020).	13.01
mws0639	2,3-Dihydroxybenzoic Acid	Organic acids	It was found to be the strongest antioxidant (Spiegel et al., 2020); inhibiting cancer cell growth in vitro (Sankaranarayanan et al., 2020)	14.42
Lmhn001580	Sinapoyltartaric acid	Phenolic acids	–	10.80

### Association analysis of the metabolome and transcriptome datasets

**Figure 6:**
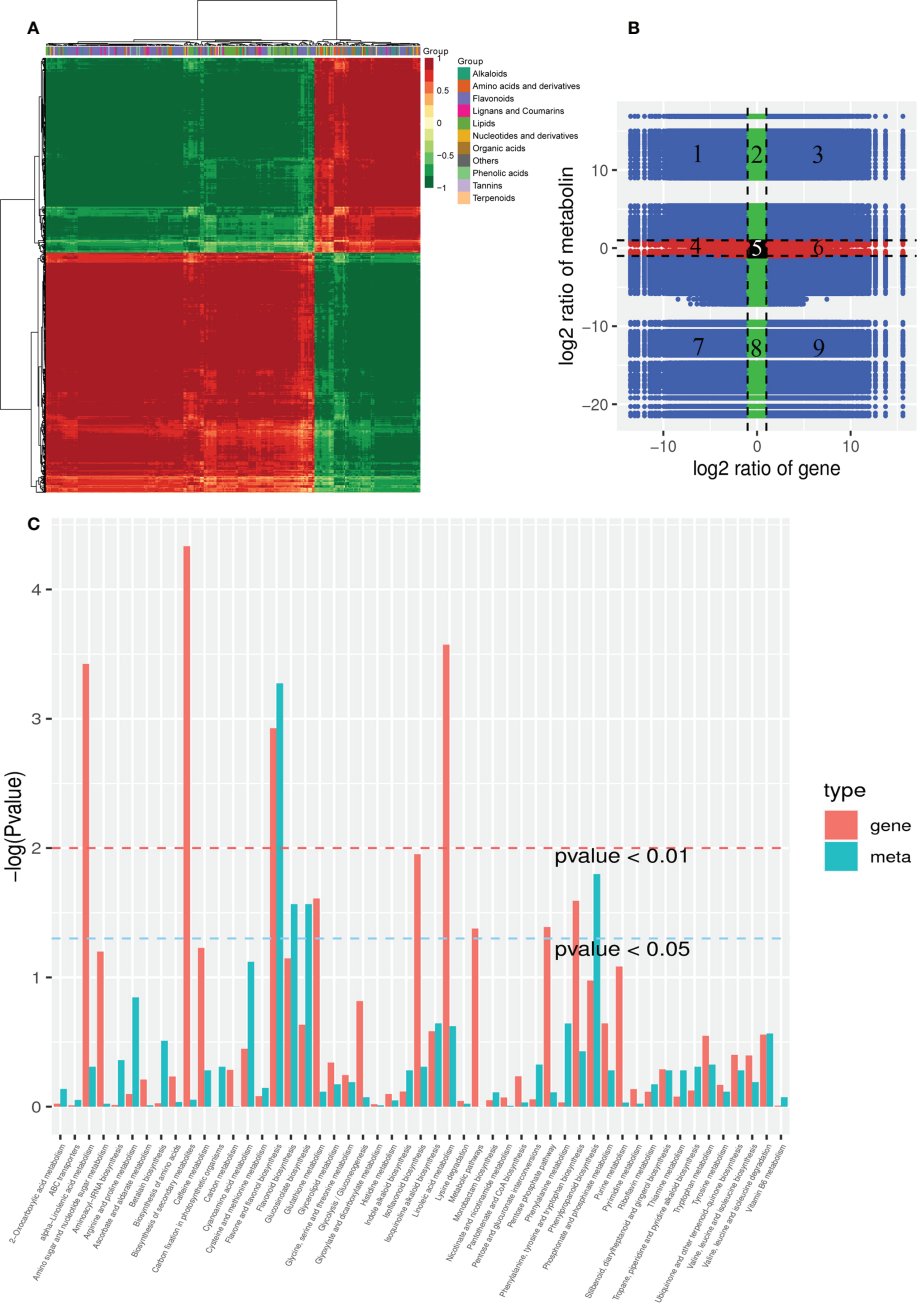
Combined analysis of the transcriptome and metabolome. **(A)** Cluster analysis of the correlation coefficients between the DEGs and DAMs. **(B)** DEG and DAM foldchanges in a nine-quadrant plot. The x-axis represents the log_2_FC of the DEGs, where FC is the foldchange in gene expression; that is, the ratio of the FPKM of OEGA/FPKM of CON. The y-axis represents the log_2_FC of DAMs, where FC is the foldchange in the metabolite content between the OEGA and CON groups. Black dots represent unchanged genes or metabolites, green dots represent DAMs vs. unchanged gene expression, red dots represent DEGs vs. unchanged metabolites, and blue dots represent changes in gene expression and metabolites. The black dashed lines represent the thresholds for log_2_FC = 1 and divide the graph into nine quadrants. The metabolites and genes in the third and seventh quadrants showed consistent changes. **(C)** Joint KEGG enrichment *P* value histogram of the DEGs and DAMs in the CON and OEGA. The smaller the *P* value, the higher the degree of enrichment.

**Figure 7:**
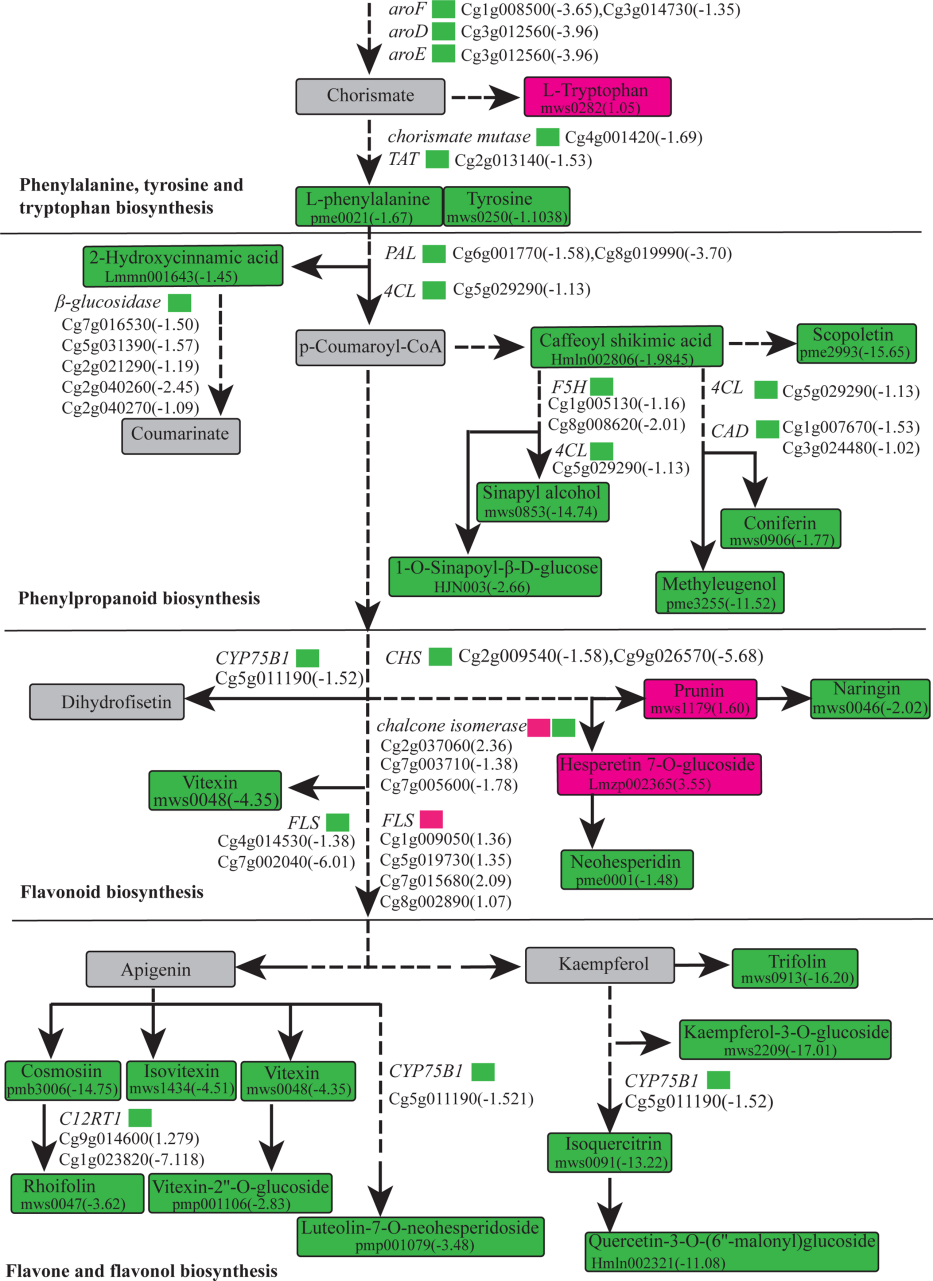
Biosynthetic pathway of the phenylpropane compounds.

A histogram was generated based on the results of the DAMs and DEGs conjoint KEGG enrichment analysis, showing the degree of enrichment of the pathways with differential metabolites and genes (**Figure 6C**). Results showed 49 co-mapped pathways involving the DEGs and DAMs (**Figure 6C**). Among these pathways, “flavone and flavonol biosynthesis” was significantly enriched, and “flavonoid biosynthesis”, “phenylpropanoid biosynthesis”, “isoquinoline alkaloid biosynthesis”, “glucosinolate biosynthesis”, “linoleic acid metabolism”, “valine, leucine and isoleucine degradation”, and “phenylalanine, tyrosine and tryptophan biosynthesis” were also enriched in the CON compared to the OEGA group (**Figure 6C**). This suggested that these metabolic pathways are activated by the overexpression of *CcGASA4*. The DEGs and DAMs involved in “flavone and flavonol biosynthesis”, “flavonoid biosynthesis”, and “phenylpropanoid biosynthesis” are shown in **Figure 7**. The accumulation of phenylpropane compounds was significantly reduced in the OEGA (**Figure 7**). When a set of genes associated with the flavonoid pathway was transcriptionally profiled, it was found that tyrosine aminotransferase (*TAT*), 4-coumarate-CoA ligase (*4CL*), chalcone synthesis (*CHS*), chalcone isomerase (*CHI*), flavanone 5-hydroxylase (*F5H*), and flavonoid 3’-monooxygenase (*CYP75B1*) were downregulated following the overexpression of *CcGASA4*. These findings were consistent with the reduced capacity of the transgenics for flavonoid synthesis.

The correlation analysis showed that there were 1,201,479 correlation pairs with |PCC| ≥ 0.8 and *P* < 0.05, including 3,515 DEGs and 294 DAMs. The correlation calculation results of the DAMs and DEGs were plotted in clustered heatmaps (**Figure 6A**). The nine-quadrant plot results showed 3,437 genes with |PCC| ≥ 0.8, and 99 metabolites were identified in the third and seventh quadrants (**Figure 6B**). Among these genes, 87 flower-related DEGs were identified with 14 metabolites in the third quadrant (**Table S11**). The correlation analysis results further showed that 125 plant–pathogen interaction-related genes had strong positive correlation coefficients (*P* < 0.05) with 14 metabolites (**Table S12**). The relative abundance levels of nine highly-accumulated OEGA metabolites were highly correlated with 23 plant hormone signal transduction-related genes (**Table S13**).

**Figure 8:**
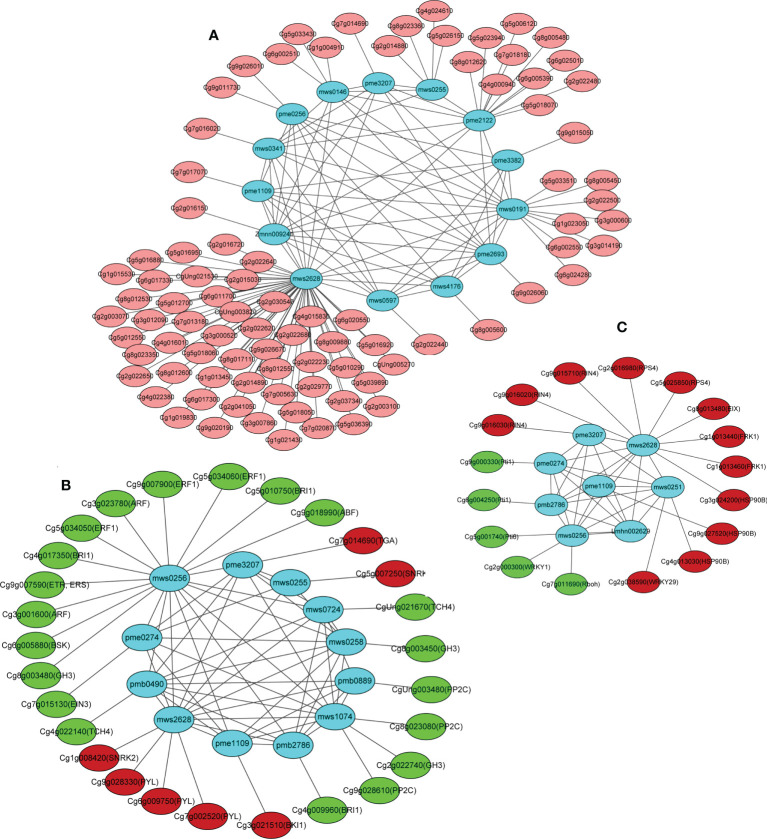
Pearson’s correlation network model diagram of the DEGs and DAMs.**(A)** Cluster I network represents differentially expressed genes and metabolites associated with flowers. **(B)** Cluster II network represents plant hormone signal transduction pathway related DEGs and DAMs. **(C)** Cluster III network represents plant pathogen resistance-related DEGs and DAMs. Each blue node (circle) represents a metabolite. Significant metabolite–metabolite and gene–metabolite connections are represented by the edges (lines). A network graph was constructed using the correlation data for the CON and OEGA metabolites. Nodes without connections to other nodes were deleted. Red (or pink) and green colors represent up- or downregulation in the CON vs. OEGA groups, respectively.

Interaction networks between the DEGs and DAMs were organized based on the PCC results. Only the correlation pairs with a correlation coefficient > 0.8 were included in the analysis. The networks revealed three separated structures containing two major networks and one small network (**Figure 8**), where the cluster I network included three nucleotides and their derivatives (cytosine, guanine, and xanthine), three amino acids and their derivatives (histamine, *N*-acetyl-L-threonine, and L-alanyl-L-phenylalanine), five alkaloids (betaine, *N*-acetylputrescine, nicotinic acid methyl ester (methyl nicotinate), 5-hydroxyindole-3-acetic acid, and citpressine I), and three organic acids (*N*-methyl-4-aminobutyric acid, 2-hydroxyisocaproic acid, and muconic acid) (**Figure 8A**). A large number of flower development-related genes in the DEGs were positively correlated with the 14 above-mentioned metabolites, such as the upregulated genes *FOREVER YOUNG FLOWER* (*FYF*; *Cg9g026060*), *EARLY FLOWERING BY OVEREXPRESSION 2* (*EFO2*; *Cg9g026670*), *ANTHESIS POMOTING FACTOR 1* (*APRF1*; *Cg3g000600* and *Cg3g000520*), and *ANTHESIS POMOTING FACTOR 1* (*COL4*; *Cg4g000940*) and downregulated genes *EARLY FLOWERING 3-like (ELF3*; *Cg1g009190*), *ANTHESIS POMOTING FACTOR 1* (*APRF1*; *Cg3g000660* and *Cg3g000700*), and *EARLY FLOWERING 1* (*ELF1*; *Cg9g019180*) (**Table S11**). The cluster II network included three organic acids (*N*-methyl-4-aminobutyric acid, muconic acid, and 6-aminocaproic acid), three nucleotides and their derivatives (cytosine, guanine, and 8-hydroxyguanosine), two amino acids and their derivatives (L-valine and L-isoleucine), two lipids (punicic acid [9Z,11E,13Z-octadecatrienoic acid] and 9-hydroxy-10,12,15-octadecatrienoic acid), one lignan and coumarin (daphnetin), and one alkaloid (p-coumaroylputrescine) (**Figure 8B**). The upregulated genes *serine/threonine-protein kinase SRK2* (*SNRK2*; *Cg5g007250* and *Cg1g008420*), *abscisic acid receptor PYR/PYL* (*PYL*; *Cg6g009750*, *Cg7g002520*, and *Cg9g028330*), *transcription factor TGA* (*TGA*; *Cg7g014690*), and *BRI1 kinase inhibitor 1* (*BKI1*; *Cg3g021510*) in the plant hormone signal transduction pathway were positively correlated with the accumulation of cytosine (mws0255), *N*-methyl-4-aminobutyric acid (mws2628), muconic acid (pme3207), and guanine (pme1109). The downregulated genes *auxin response factor* (*ARF*; *Cg3g001600* and *Cg3g023780*), *protein brassinosteroid insensitive 1* (*BRI1*; *Cg4g017350*, *Cg5g010750*, and *Cg4g009960*), *ethylene-responsive transcription factor 1* (*ERF1*; *Cg5g034050*, *Cg5g034060*, and *Cg9g007900*), *BR-signaling kinase* (*BSK*; *Cg6g005880*), *auxin responsive GH3 gene family* (*GH3*; *Cg8g003480*, *Cg8g003450*, and *Cg2g022740*), *ethylene receptor* (*ETR*; *Cg9g007590*), *ABA responsive element binding factor* (*ABF*; *Cg9g018990*), *ethylene-insensitive protein 3* (*EIN3*; *Cg7g015130*), *protein phosphatase 2C* (*PP2C*; *Cg8g023080*, *Cg9g028610*, and *CgUng003480*), and *xyloglucosyl transferase TCH4* (*TCH4*; *Cg4g022140* and *CgUng021670*) in the plant hormone signal transduction pathway were positively correlated with the accumulation levels of L-valine (mws0256), 6-aminocaproic acid (pme0274), L-isoleucine (mws0258), daphnetin (mws1074), p-coumaroylputrescine (pmb0490), punicic acid (9*Z*,11*E*,13*Z*-octadecatrienoic acid) (pmb0889), 9-hydroxy-10,12,15-octadecatrienoic acid (pmb2786), and 8-hydroxyguanosine (mws0724) (**Figure 8B**). The cluster III network included two organic acids (*N*-methyl-4-aminobutyric acid and muconic acid) and two nucleotides and derivatives (thymine and guanine). These four metabolites were highly accumulated in the OEGA group (**Table S9**). The plant pathogen resistance-related genes *senescence-induced receptor-like serine/threonine-protein kinase* (*FRK1*; *Cg1g013440* and *Cg1g013460*), *disease resistance protein RPS4* (*RPS4*; *Cg2g016980* and *Cg5g025850*), *heat shock protein 90kDa beta* (*HSP90B*; *Cg3g024200*, *Cg4g013030*, and *Cg9g027520*), *ethylene-1,4-beta-xylanase* (*EIX*; *Cg8g013480*), *RPM1-interacting protein 4* (*RIN4*; *Cg9g015710*, *Cg9g016020*, and *Cg9g016030*), and *WRKY transcription factor 29* (*WRKY29*; *Cg2g038590*) were positively correlated with the content of *N*-methyl-4-aminobutyric acid, thymine (mws0251), muconic acid, and guanine (**Figure 8C**). The resulting network map indicated that *CcGASA4* could regulate the co-expression of DEGs and DAMs associated with plant–pathogen interactions and plant hormone signal transduction.

## Discussion

The accumulation of secondary metabolites in plants is not only affected by external environmental factors, such as temperature, light, and metal ions (Ramakrishna and Ravishankar, 2011), but also by plant genetic factors. In terms of the functions of *GASA* genes, recent studies on various monocotyledonous and dicotyledonous plant species have assessed the model plants rice and *Arabidopsis*, respectively (Nahirñak et al., 2012). To date, few functional studies on the GASA family have been conducted in fruit trees to explore the function of *GASA* genes using integrated transcriptomic and metabolomic analyses. We therefore used the previously-cloned *CcGASA4* gene to develop OEGA lines and study the effects of CcGASA4 on the intrinsic metabolism and transcription of pomelo trees.

Flavonoids comprise a large class of ubiquitous and versatile secondary metabolites that are synthesized through a branch of the phenylpropanoid metabolic pathway in plants (Lewis et al., 2011). Flavonoids have diverse functions, including defense against phytopathogens, protection against UV light damage and oxidative stress, and the regulation of auxin transport and allelopathy (Lewis et al., 2011). Our metabolomic data suggest that the total flavonoid content in the CON group was higher than that in the OEGA, which may have been due to the decreased synthesis of phenylalanine in OEGA. Chorismate is a common precursor for the downstream synthesis of L-tryptophan, phenylalanine, and tyrosine. We found an increased accumulation of L-tryptophan and decreased accumulation of L-tyrosine and L-phenylalanine, which suggested a stronger metabolic flow to the L-tryptophan biosynthesis branch. The expression levels of the chorismate mutase-encoding gene Cg4g001420, which is a key enzyme that controls the biosynthesis of the phenylalanine and tyrosine branches, were significantly downregulated in OEGA. Therefore, the overexpression of *CcGASA4* decreased the precursor supply for flavonoid biosynthesis by varying the substrate distribution in the aromatic amino acid biosynthetic pathway.

Previous studies have mainly focused on the transcriptional regulation of flavonoid metabolism. Flavonoids are regulated by various transcription factors from the R2R3-MYB family, such as *VvMYB4* in grapevines (Pérez-Díaz et al., 2015), *AtMYB7* in *Arabidopsis thaliana* (Fornalé et al., 2014), *FaMYB1* in strawberries (Aharoni et al., 2010), and *CmMYB8* in chrysanthemums (Zhu et al., 2020). The overexpression of CcGASA4 was accompanied by a reduced abundance of transcripts generated from the phenylpropanoid pathway genes *aroF*, *aroD*, *aroE*, *PAL*, *TAT*, and *4CL*, suggesting that CcGASA4 is an inhibiting factor in flavonoid synthesis. Decreased phenylpropanoid pathway genes were a major cause of the decreased tissue content of several flavonoid compounds, including rutin, quercetin, trifolin, vitexin, and rhoifolin, in the *CcGASA4*-overexpressing plants. Similar results have been reported in other species. For example, the overexpression of *CmMYB8* has been found to result in significantly-reduced flavonoid and lignin contents in transgenic chrysanthemums (Zhu et al., 2020). *DETF* (*TRINITY_DN130563_c0_g3*) has high homology with *CmMYB8* in chrysanthemums, and its expression levels have been found to be the opposite of its accumulation in flavonoids (Zou et al., 2021). Therefore, we speculate that the functional role of CcGASA4 is similar to that of these transcription factors and potentially inhibits the biosynthesis of flavonoids in Shatian pomelo.

Glycosylation, which is a typical modification of flavonoids, usually alters the stability, solubility, and activity of flavonoids and facilitates their transport to the vacuole for long-term storage (Bowles et al., 2006). There were 16 significantly-enriched flavonoids in the OEGA plants, eight of which existed in the form of glucosides. The attachment of sugar groups to flavonoid or isoflavonoid compounds is catalyzed by members of large families of enzymes, such as uridine diphosphate glycosyltransferases (UGTs) (Bowles et al., 2006). Our data showed that the *UGT72Es* Cg1g003710 and Cg1g003720 were upregulated by 7.305- and 2.878-fold, respectively, compared to those in the CON plants. This indicates that the high accumulation of these glycosylated flavonoids may be caused by the upregulation of *UGT72E* in CcGASA4-overexpressing plants. However, the regulatory mechanisms of action of *CcGASA4* in flavonoid biosynthesis require further investigation.

Among the DAMs in the OEGA plants, the flavonoid metabolite dihydrokaempferol-7-O-glucoside was the most significantly altered. Dihydrokaempferol-7-O-glucoside exhibits flower-promoting activity (Nakanishi et al., 1995) and may participate in fruit development in Osage orange fruits (Tian et al., 2006). The co-enrichment network showed that many flower-related genes were induced by *CcGASA4* (**Figure 8A**). Our previous study found that *CcGASA4* is mainly expressed in citrus flowers (Wu et al., 2020) and that the *CcGASA4* promoter regulates the expression of the *GUS* gene in *Arabidopsis* flowers (data not published). *CcGASA4* may therefore promote flower development by regulating the expression of various flower-related genes and accumulation of dihydrokaempferol-7-O-glucoside.

The overexpression of *CcGASA4* further reduced the tissue content of free fatty acids, lyso-phosphatidylethanolamine, lysophosphatidylcholine, and glycerol ester. It also led to the downregulation of many genes encoding components of lipid synthesis. *CcGASA4* may therefore regulate lipid synthesis. Lipids are the core components of plant cell membranes and provide structural integrity and energy for various metabolic processes (Fischer et al., 2004). Sub-cellular experiments have demonstrated that CcGASA4 is restricted to the nucleus and plasma membrane (Wu et al., 2020), which suggests that *CcGASA4* may utilize lipids in large quantities and result in lower lipid levels in cells.

The transcriptome data also showed that a large number of DEGs were enriched in the plasma membrane-bound cell projections. GO enrichment analysis showed that the DEGs were also significantly enriched in hormone metabolic processes, responses to SA, and responses to ABA (**Figure 2A**). This indicates that CcGASA4 may have various biological functions on the cell membrane and is likely to be involved in multiple hormone signaling pathways. This is consistent with our previous findings, which showed that CcGASA4 localizesd to the nucleus and plasma membrane, and respondsed to exogenous hormones (Wu et al., 2020). Additionally, several auxin signaling pathway genes, such as *ARF* and *GH3*, were significantly downregulated in the OEGA group (**Figure 8C**). The auxin signaling pathway regulates cell enlargement (Cleland, 1971). *BRI1*, *BSK*, and *TCH4* were also significantly downregulated and are downstream of the BR signaling pathway, which can promote plant cell elongation. In contrast, *BKI1*, which exerts antagonistic effects with *BRI1*, was significantly upregulated (**Figure 8C**). We further observed that the cytokinin metabolic pathway regulated cell division and shoot initiation. The *CRE1* gene, which is located downstream of this pathway, was significantly upregulated. Additionally, we found that the expression of *GID2* was upregulated (**Figure 8C**) and that this gene is an inhibitor of the downstream *DELLA* gene in the gibberellin signaling pathway that promotes stem elongation. This evidence suggests that *CcGASA4* promotes cell division and inhibits cell enlargement, elongation, and stem elongation. We also observed the downregulated expression of *ETR*, *EIN3*, and *ERF1/2* in the downstream regulatory pathway of ethylene, which promotes fruit ripening and senescence (**Figure 8C**). ABA can promote stomatal closure and seed dormancy through signaling, whereas the expression of *ABF*, which is located downstream of this signaling pathway, was significantly inhibited (**Figure 8C**). This indicates that GASA4 may also inhibit fruit senescence and ripening. It has further reported that FaGAST, which is a member of the GASA family in strawberries, can arrest cell elongation during fruit ripening. Ectopic expression of *FaGAST* from *Fragaria vesca* in *Arabidopsis thaliana* has been found to delay the growth rate and reduce the sizes of the fruits (de la Fuente et al., 2006).

In the current study, we found that the OEGA plants had higher expression levels of disease resistance-related genes than the CON plants did. Several resistance-related genes, including *FRK1* (*Cg1g013440* and *Cg1g013460*), *RIN4* (*Cg9g015710* and *Cg9g016020*), *HSP90* (*Cg3g024200*), and *RPS4* (*Cg2g016980* and *Cg5g025850*), were identified as DEGs. Of the 25 OEGA-specific accumulated metabolites, 10 (six flavonoids, one alkaloid, one terpenoid, one organic acid, and one other) were involved in disease resistance (**Table 1**). The functions of these metabolites are as follows: luteolin-4’-O-glucoside and skullcapflavone II have anti-inflammatory properties; acacetin-7-O-galactoside, kokusaginine, and betulin have anti-cancer properties; 2,3-dihydroxybenzoic acid can inhibit cancer cell growth *in vitro* and has antioxidant properties; taxifolin-3’-O-glucoside participates in the treatment of BPH; 5-hydroxy-6,7,8,3’,4’-pentamethoxyflavone is involved in anti-basophilic leukemia in rats; 5-hydroxy-6,7,3’,4’-tetramethoxyflavone protects hepatocytes; and ribitol participates in abiotic reactions. In addition, the flavonoid 3’,4’,7-trihydroxyflavone can not only prevent apoptotic cell death in neuronal cells from hydrogen peroxide-induced oxidative stress, but also has antibacterial properties (Kwon et al., 2015; Dzotam et al., 2018). The flavonoids acacetin-7-O-glucoside and 1,3,7-trimethoxyxanthone also have antibacterial properties (Matsushima et al., 1985; Shen et al., 2004). Through the association analysis (**Figure S5**), seven genes related to disease resistance were found to be significantly associated with these disease-resistant metabolites. Therefore, the high expression of disease resistance-related genes in the OEGA plants and the higher content of flavonoids and other metabolites may improve the disease resistance of transgenic plants.

## Conclusions

In this study, we investigated the positive role of *CcGASA4* in citrus plants. Novel regulatory and functional candidates in plant–pathogen interactions, plant hormone signal transduction, and flavonoid and flavonol metabolism were successfully identified through comparative transcriptional and metabolic analyses. Our findings confirm that *CcGASA4*, which is a GASA family gene in *Citrus*, inhibits the synthesis of lignin and flavonoids and may play a role in plant–pathogen interactions, inhibiting growth, and affecting flowering development. Future studies should test the effects of CcGASA4 on resistance to bacterial and fungal infections and on the growth and development of citrus plants, all of which comprise traits that are important for increasing the yield of *Citrus.*

## Data availability statement

The datasets presented in this study can be found in online repositories. The name of the repository and accession number can be found below: BioProject, accession number PRJNA884542.

## Author contributions

TW conducted the experiments and wrote the manuscript. KL revised the manuscript. MC helped perform qRT-PCR. QG helped to adjust the picture format. BJ completed the logical revision of some contents. YZ provided guidance throughout the study. All authors contributed to the article and approved the submitted version.

## Funding

This research was financially supported by the Key-Area Research and Development Program of Guangdong Province (2022B0202070002), Guangdong Provincial Science and Technology Special Project in 2021 (210907114532092), National Natural Sciences Foundation of China (32002016), and Guangdong Provincial Special Fund for Modern Agriculture Industry Technology Innovation Special Teams (2022KJ108).

## Acknowledgments

The authors thank Dr. Yanyan Ma and Dr. Chunzhen Cheng for their support with the experiments.

## Conflict of interest

The authors declare that the research was conducted in the absence of any commercial or financial relationships that could be construed as a potential conflict of interest.

## Publisher’s note

All claims expressed in this article are solely those of the authors and do not necessarily represent those of their affiliated organizations, or those of the publisher, the editors and the reviewers. Any product that may be evaluated in this article, or claim that may be made by its manufacturer, is not guaranteed or endorsed by the publisher.
