# Supplementary figures and images for: Transcriptome and metabolome analyses of Shatian pomelo (Citrus grandis var. Shatinyu Hort) leaves provide insights into the overexpression of the gibberellin-induced gene CcGASA4
Source: Front Plant Sci. 2022 Nov 3;13:1022961. doi: 10.3389/fpls.2022.1022961 (PMC9671072; doi:10.3389/fpls.2022.1022961)

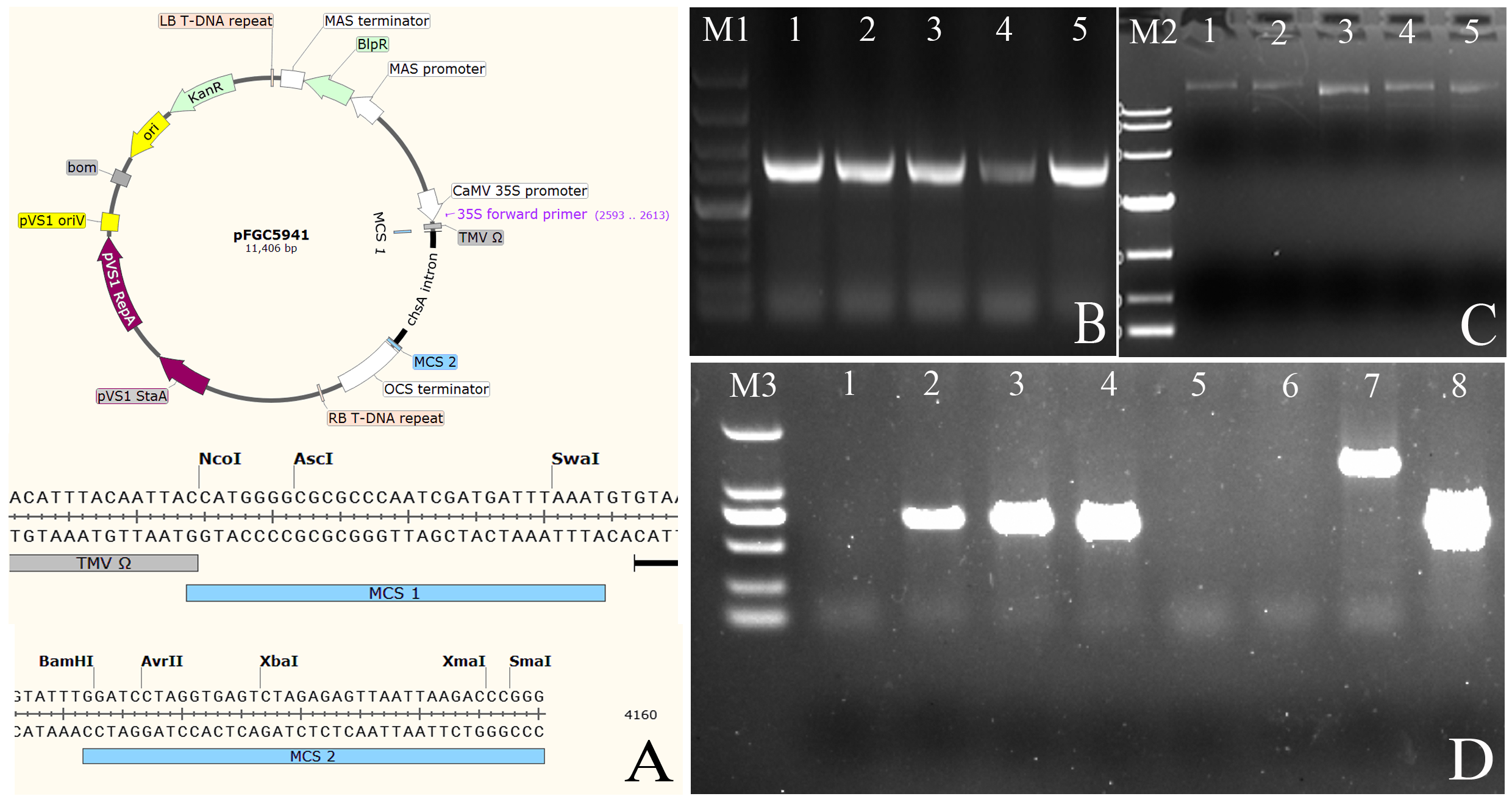

Supplement: Supplementary file 1 [file Image_1.tif]

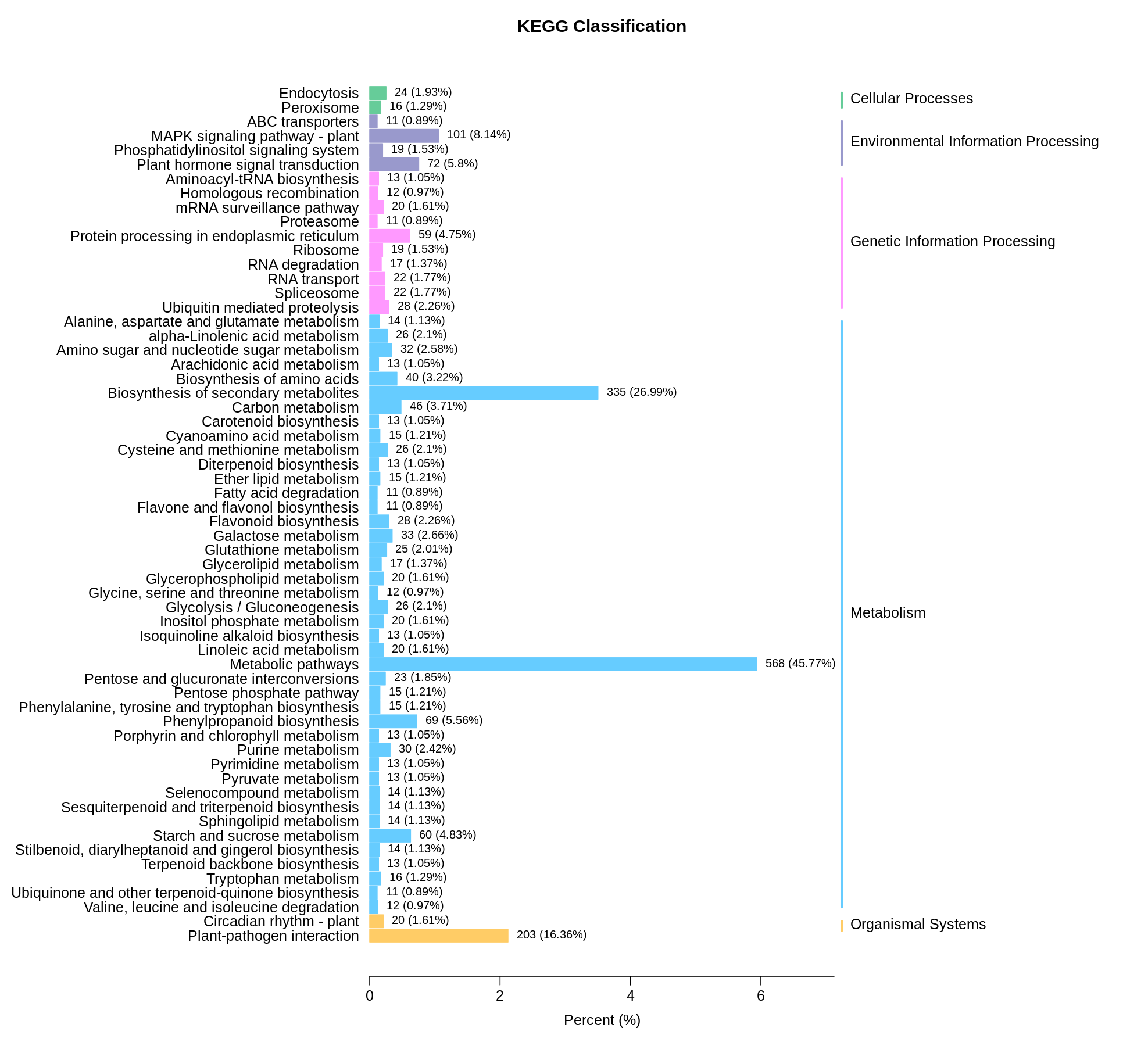

Supplement: Supplementary file 2 [file Image_2.tif]

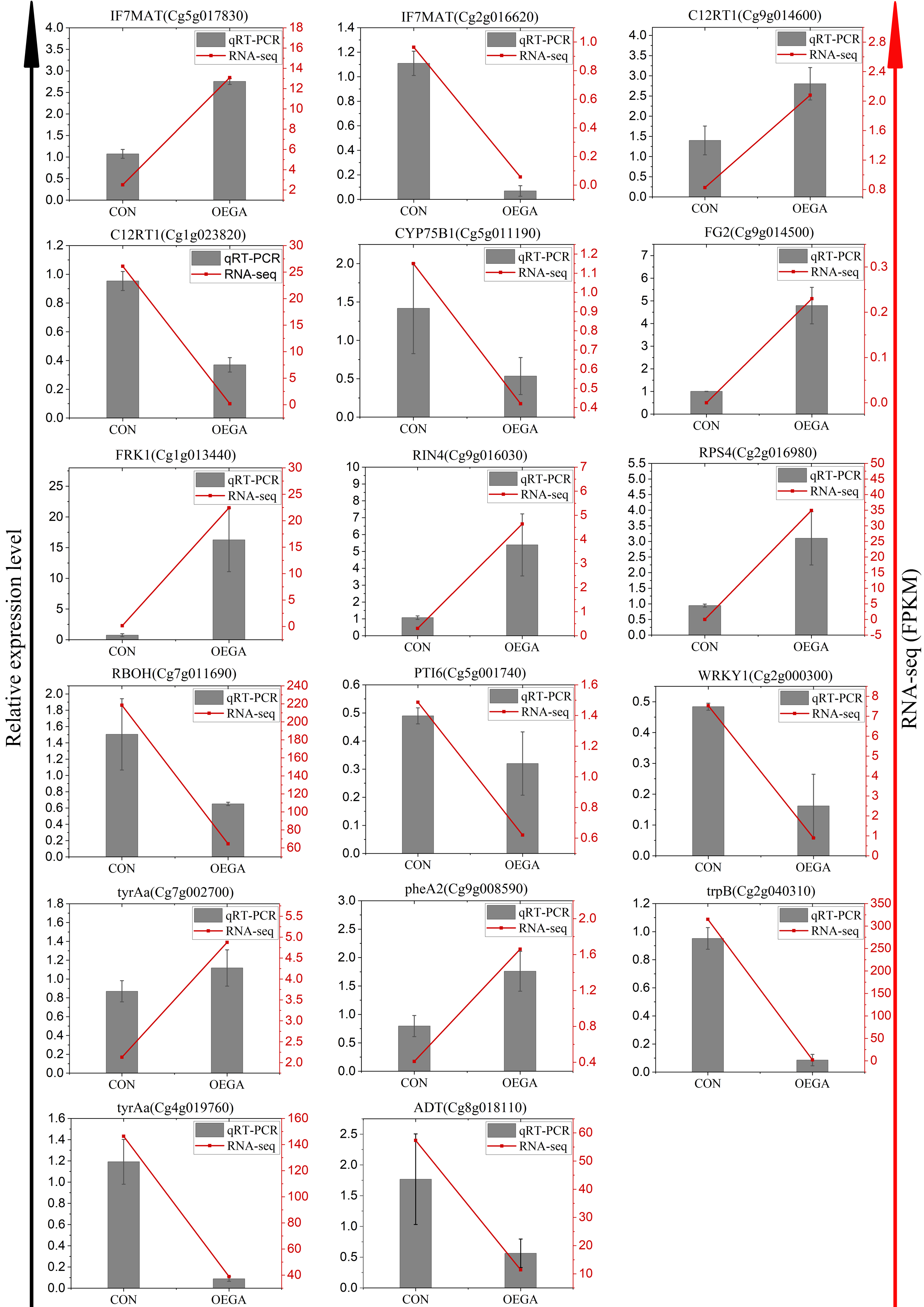

Supplement: Supplementary file 3 [file Image_3.tif]

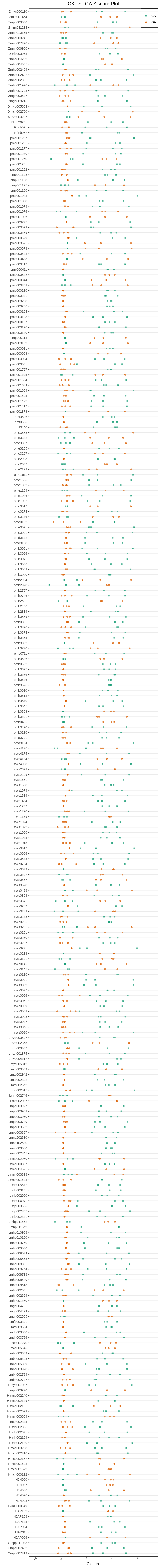

Supplement: Supplementary file 4 [file Image_4.tif]

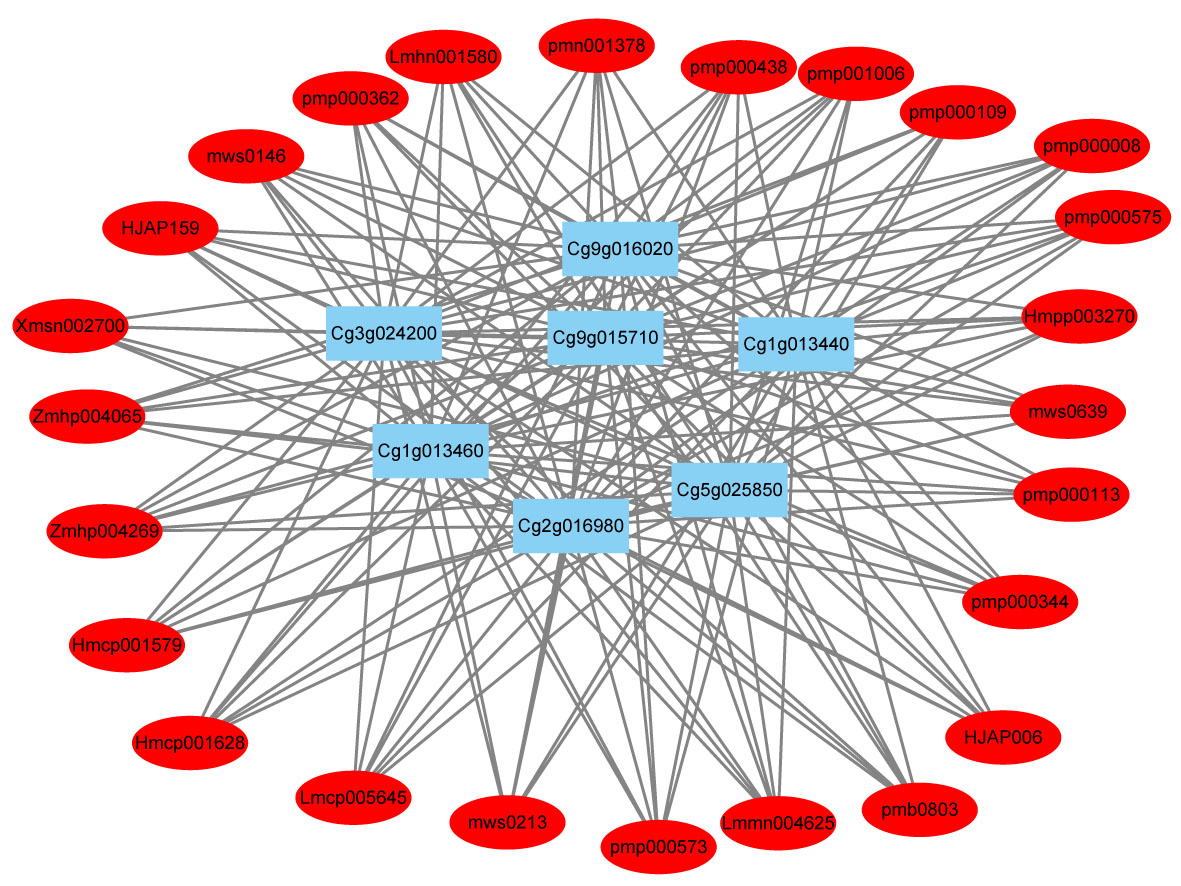

Supplement: Supplementary file 5 [file Image_5.tif]
